# Multisensory action effects facilitate the performance of motor sequences

**DOI:** 10.3758/s13414-020-02179-9

**Published:** 2020-11-01

**Authors:** Mengkai Luan, Heiko Maurer, Arash Mirifar, Jürgen Beckmann, Felix Ehrlenspiel

**Affiliations:** 1 Department of Sport and Health Sciences, Chair of Sport Psychology, Technische Universität Munich, Campus D - Georg-Brauchle-Ring 60/62, 80992 Munich, Germany; 2 Neuromotor Behavior Laboratory, Institute of Sport Science, Justus Liebig University, Giessen, Germany; 3 School of Human Movement and Nutrition Sciences, University of Queensland, Brisbane, Australia

**Keywords:** Motor sequence, Multisensory, Action effect, The ideomotor principle

## Abstract

Research has shown that contingent, distinct action effects have a beneficial influence on motor sequence performance. Previous studies showed the beneficial influence of task-irrelevant action effects from one modality (auditory) on motor sequence performance, compared with no task-irrelevant action effects. The present study investigated the influence of task-irrelevant action effects on motor sequence performance from a multiple-modality perspective. We compared motor sequence performances of participants who received different task-irrelevant action effects in an auditory, visual, or audiovisual condition. In the auditory condition, key presses produced tones of a C-major scale that mapped to keys from left to right in ascending order. In the visual condition, key presses produced rectangles in different locations on the screen that mapped to keys from left to right in ascending order. In the audiovisual condition, both tone and rectangle effects were produced simultaneously by key presses. There were advantages for the audiovisual group in motor sequence initiation and execution. The results implied that, compared with unimodal action effects, action effects from multiple sensory modalities can prime an action faster and strengthen associations between successive actions, leading to faster motor sequence performance.

**Supplementary Information:**

The online version contains supplementary material available at 10.3758/s13414-020-02179-9.

When we act, we usually have a goal in mind—we write a manuscript with the goal of publishing our research, we reach for a glass of wine with the goal of drinking it, and we press “k” on the keyboard with the goal of it appearing on the monitor. The idea that these goals drive our actions—even motor actions—is the central tenet of the ideomotor principle (for a review, see Stock & Stock, 2004). Motor actions are generated to achieve desired goals, which are to bring intended and expected sensory effects (Hommel, 1996). Thus, sensory action effects are important parts of an action’s mental representation (Kunde, Koch, & Hoffmann, 2004). Learning or performing a particular action requires the acquisition of associations between actions and their effects (Elsner & Hommel, 2001). Previous research has shown that anticipation of sensory action effects can prime the action (Kunde, 2001; Kunde et al., 2004), and also plays a crucial role in the acquisition and execution of motor sequences (Hoffmann, Sebald, & Stöcker, 2001; Stöcker & Hoffmann, 2004; Stöcker, Sebald, & Hoffmann, 2003). Previous studies on the influence of sensory action effects on motor sequence performance focused on the influence of a unimodal action effect. However, to our knowledge, the role of action effects from multiple sensory modalities perspective has hardly been investigated.

According to the ideomotor principle, sensory action effects are integrated parts of action representation, and an action is bidirectionally associated with action effects (Greenwald, 1970). Therefore, the sensory effects of an action may “prime” the execution of the action, if bidirectional associations between actions and their effects have been acquired. In a study by Elsner and Hommel (2001), during an acquisition phase, participants were free to press either the left or right key in response to a centrally presented visual signal. Each key, however, contingently triggered either a high or low tone. In the test phase, participants were asked to respond to these tone signals by pressing one of the same two keys. Participants responded faster to a tone if they had to press the key that had previously triggered that tone, compared with the other key. Further, when asked to freely choose which key to press in response to a tone, participants preferred to press the key that had previously triggered that tone.

More straightforwardly related to the assumption that anticipation of effects generates action, studies have also investigated whether predictable sensory effects still have an impact on an action despite following the action, and thus being retroactive. Studies on the response-effect compatibility have repeatedly shown this to be the case. For example, in a four-choice reaction time paradigm, participants responded faster when the responses’ location spatially corresponded to the location of the responses’ visual effects (one of four horizontally aligned boxes lit up on a monitor; task-irrelevant) than when locations did not spatially correspond. Additionally, in a two-choice task, participants initiated a certain force faster when the action triggered auditory effects of corresponding, rather than noncorresponding, intensity (Kunde, 2001). The response-effect compatibility effect has been reported for many task variations and relations between responses and effects (Koch & Kunde, 2002; Kunde, 2001, 2003; Kunde et al., 2004). Thus, although sensory effects are technically irrelevant when the actor only has to respond to a trigger stimulus and the effects follow this response, the effects or their anticipation still serve a function in selecting the response (Hommel, 2009).

Given that a significant amount of everyday behavior is structured in sequential succession, and our actions sequentially interact with effects in the environment, the role of anticipation of action effects has also been studied in relation to learning of movement sequences. Learning of movement sequences is often investigated within a serial reaction task (SRT) paradigm, wherein participants are asked to respond to successively presented stimuli, and each response triggers the next stimulus presentation. Hoffmann et al. (2001) found that task-irrelevant sensory effects from auditory modality, such as tone effects, influence the serial learning in SRT. The reaction time to ordered stimuli was faster for the experimental group with tone effects than for the control group without tone effects if tone effects irrelevant to the task were contingently mapped to the responses. Based on such findings, it has been argued that stable associations not only develop between responses and their effects but also between the successive effects themselves (Greenwald, 1970). Greenwald (1970) postulated that for sequence control, eventually the representation of the sequence of (anticipated) effects takes over response control. After repeatedly experiencing stimulus–response–effect triplets, sensory effect production leads to the anticipation of the next effect, which in turn triggers the next response. This process can be viewed as effect chaining; however, its consequences are less visible in the classic SRT task, as each response (also) occurs in response to a stimulus.

If an entire sequence needs to be learned and reproduced—for example, a piano melody—effect chaining should be more evident. In a study by Stöcker and Hoffmann (2004), participants learned two motor sequences: a short sequence of three ordered letters and a long sequence of six ordered letters. In one group of participants (“tone group”), each key press was followed by an immediate tone effect that was distinctively and contingently mapped to the key press. The tones were of the C-major scale and mapped to the keys from left to right, in ascending order. The other group of participants received no auditory effects (“no-tone group”). The two sequences were learned within an SRT paradigm, with each letter of a sequence first presented as a stimulus on a computer monitor, to which participants then responded, resulting in a contingent effect (or none), and after a short interval, the next letter of the sequence was presented. Additionally, a label on the screen always indicated which of the two sequences was presented. Performance was assessed within a choice reaction time paradigm. After the label indicating which sequence was to be executed (the short or the long sequence) appeared on the screen, participants were to correctly reproduce the whole sequence as quickly as possible. It was assumed that, compared with the no-tone group, effect chaining in the tone group would not only lead to faster reaction times but also facilitate associations of successive elements in the motor sequence and the chunking of the elements of the sequence into a larger unit. Based on the general finding that motor sequences with fewer elements are initiated faster than motor sequences with more elements (Verwey, 1999), the initiation times (ITs) of motor sequences would presumably be smaller in the tone than in the no-tone group. Results showed both motor sequences were initiated significantly faster in the tone group. This effect is associated with shorter interresponse times (IRTs; i.e., the transition between keys within a sequence) in the tone group than no-tone group. These findings supported that action–effect associations lead to faster initiation and execution of motor sequences.

Stöcker and Hoffmann (2004) showed a beneficial influence of the task-irrelevant action effects from one modality (auditory), compared with no auditory effects. The action effects in the original Stöcker and Hoffmann paradigm consisted of kinesthetic feedback from the fingers and proprioceptive perception of responses (Greenwald, 1970). When they are augmented by contingent sensory effects from an auditory modality (i.e., tones), all these sensory effects form the coherent action effects. The action effects from different modalities are coded into the action representation as different features of an event file in a distributed fashion (Hommel, 2004). These multimodal features are becoming effective retrieval cues or primes of the associated movement pattern. Numerous studies (e.g., Ladwig, Sutter, & Müsseler, 2013; Sedda, Monaco, Bottini, & Goodale, 2011; Zmigrod, Spapé, & Hommel, 2009) have provided evidence for interactions between features from different sensory modalities and between multisensory features and actions. Action effects from different modalities interact with each other, making the associations between action and effect and between successive actions appear to grow stronger (Kunde et al., 2004; Stöcker & Hoffmann, 2004). It may be speculated that the more action effect features are present and anticipated, the greater the activation of the representation of actions and the stronger the associations between successive actions. If this were the case, task-irrelevant action effects from multiple sensory modalities could prime the action more efficiently and make the associations between successive actions stronger than task-irrelevant action effects from a single sensory modality. Specifically, the initiation and execution of motor sequences would be faster in a condition with task-irrelevant action effects from multiple sensory modalities.

Thus, the purpose of the present study was to investigate the role of task-irrelevant action effects from a multisensory perspective and test whether there is an advantage for bimodal action effects, compared with unimodal action effects. The experimental design in this study was nearly identical to Stöcker and Hoffmann’s (2004) paradigm, with the exception that different action effects were used. We compared motor sequence performance of participants who received different action effects in an auditory, visual, or audiovisual condition. Each participant practiced two motor sequences (short and long). The mapping of task-irrelevant action effects to key presses differed in each group. In the auditory condition, key presses produced tones of a C-major scale mapped to keys from left to right in ascending order (identical to the tone effects in Stöcker and Hoffmann’s, 2004, paradigm). In the visual condition, key presses produced rectangles in different locations on the monitor mapped to keys from left to right in ascending order. In the audiovisual condition, both tone and rectangle effects were produced simultaneously by key presses. Initiation times (ITs) and mean interresponse times (mean IRTs) were measured as indicators for motor sequence performance. Action effects features in the audiovisual condition contained more action effect features than in the auditory or visual conditions. If task-irrelevant action effects from multiple modalities indeed prime actions more efficiently and strengthen associations between successive actions, the ITs and mean IRTs should be shorter in the audiovisual condition than in the other two conditions.

## Methods

### Participants

Sixty-seven healthy students between the ages of 18 and 26 years (*M* = 24.5 years, *SD* = 2.4 years; 32 men) took part in the experiment for extra credit. Due to reasons explained below, seven of participants were replaced. Three participants were replaced in the auditory group, two were replaced in the visual group, and two were replaced in the audiovisual group. Ultimately, each group consisted of 20 participants. All participants were right-handed with either normal or corrected-to-normal vision and normal hearing. Informed written consent was obtained from all participants prior to the experiment.

Apparatus and action effect

Stimuli were presented to participants on a 17-inch monitor using MATLAB 2017b, Psychtoolbox-3 controlled by a PC. The monitor was black and the instructions were in white, 20-point Times New Roman font. The spatial resolution of the monitor was set to 1,024 × 768 and the refresh rate was 60 Hz. The viewing distance was approximately 60 cm. An ASIO compatible sound card (LOGILINK PCI-Express 7.1) was used for high precision auditory timing. The output latency of the sound card was 5 ms. Participants rested their index, middle, and ring fingers of both hands on six keys (“s,” “d,” and “f” for the left hand, and “j,” “k,” “l” for the right hand) of a German QUERTZ-keyboard throughout the experiment. When a participant pressed any of the six keys, the respective response-effect associated with the key was immediately presented. In the auditory group, 80 ms of tones of a C-major scale (“c,” “d,” “e,” “f,” “g,” and “a”) were assigned to keys in ascending order from left to right. After a key press, the corresponding tone at an intensity of 60 dB (SPL) was immediately presented from two speakers positioned on the left and right sides of the monitor. In the visual group, the response keys were associated with six yellow rectangles (width: 79-pixel, height: 153-pixel). The rectangles appeared equally spaced on a vertically centered line, with the horizontal position assigned to the keys from left to right in ascending order without overlap, and each key press triggered the corresponding rectangle to flash on the monitor for 80 ms. In the audiovisual group, each key press simultaneously produced the key-specific tone effect of the auditory group and the key-specific rectangle effect of the visual group. The action effect (tones or/and rectangles) was presented as soon as the corresponding key was pressed.

Procedure

The instructions for each experimental phase were displayed as text on the screen at the beginning of each phase. Throughout the experiment, participants placed their left index, middle, and ring fingers on the “f,” “d,” and “s” keys, and placed their right index, middle, and ring fingers on the “j,” “k,” and “l” keys.

The first phase was a short introductory phase (Phase 1), during which participants could get used to the action–effect relations by freely pressing the response keys and observing the key-press effects. The phase ended automatically after 120 seconds, or participants could end it whenever they wanted by pressing the spacebar. Usually, participants spent approximately 90–120 seconds on this phase.

In the second phase of the experiment (Phase 2), participants performed an SRT task with randomly ordered stimuli. One of the six letters was randomly presented in white, 20-point Times New Roman font at the center of the screen, and participants were asked to react as quickly as possible by pressing the key contingent to the stimulus. The corresponding action effect (tone, rectangle, or both), depending on the group, appeared when a key was pressed regardless of whether the response was correct. When an incorrect key was pressed, the word “Error” in a red, 20-point Arial font was presented for 50 ms at the bottom of the screen. The second phase consisted of two blocks of 60 trials each, and the response-to-stimulus interval (RSI) was set to 800 ms.

In the practice phase (Phase 3), participants were asked to learn two sequences that were labeled “X” and “Y.” Sequence “X” was a short sequence consisting of three ordered letters (j-s-k), and sequence “Y” was a long sequence consisting of six ordered letters (s-j-f-k-d-l). In a typical trial in the practice phase, after the presentation of a white fixation cross for 1,500 ms in the middle of the screen, the sequence-specific cue (X or Y) was displayed at the center of the upper third of the screen (above the location of the boxes in the visual and audiovisual groups) and remained on the screen throughout each trial. The first letter of the sequence was simultaneously presented at the center of the screen. When a participant correctly pressed the corresponding key, the corresponding action effect (tone, rectangle, or both) appeared. After an RSI of 800 ms, the next stimulus was presented. This manipulation was to prevent participants from practicing very fast motor sequence production during this phase. When an incorrect key was pressed, the word “Error” was presented at the bottom of the screen for 50 ms in red, 20-point Arial font. However, an incorrect response always produced the action effect contingent on the pressed key. After completing the sequence, the sequence cue disappeared, and the next trial started. The practice phase consisted of two blocks containing 30 “X” sequence trials and 30 “Y” sequence trials. The sequence trials were presented in a randomized order across each block. After finishing each block, the error rate of the block was shown on the screen for 5 s. Participants were urged to concentrate on learning the sequences properly and not only respond to key-specific stimuli, since they would later have to reproduce the sequences based on sequence cue alone, without key-specific stimulus. They were also asked to focus more on accuracy than response speed.

In the test phase (Phase 4), participants were informed that speed and accuracy were now equally important for good performance. In each trial, only the sequence-specific cue (“X” or “Y”) was presented after a white fixation cross was shown for 1,500 ms, after which participants were to type the whole sequence as quickly as possible. Each key press still produced the assigned action effect, regardless of whether the key press was correct. When an incorrect key press was made, the word “Error” flashed for 50 ms in red, 20-point Arial font at the same position as in the practice phase. Then, the fixation cross appeared, and the next trial started. Phase 4 consisted of six blocks of 60 sequence trials (30 “X” and 30 “Y”). The sequence trials were in a randomized order in each block. The error rate and mean IT of sequence typing for the block were shown on the screen for 5 s at the end of each block. There was a 30-second break between blocks. After the break, the next block could be started by pressing any key. For each trial, IT and IRTs were measured. IT was defined as the time between the onset of the sequence cue and the initial key press. IRTs were defined as the intervals between two contiguous key presses. IT and IRTs of trials with any errors were excluded from further analysis.

Data analysis

In the precursor study, Stöcker and Hoffmann (2004) did not report an effect size in their study; however, the effect size *f* can be calculated as 0.4 based on the statistical results and the number of participants in each group in this precursor study (in the precursor article, Stöcker & Hoffmann, 2004, showed that the ITs and mean IRTs for motor sequences in the group with auditory action effects were faster than the group without auditory action effects). The analysis of variance (ANOVA) of ITs data yielded *F*(1, 38) = 8.69, *p* < .01, which translates to an effect size (*f*) of 0.48. And the ANOVA of mean IRTs data yielded *F*(1, 38) = 6.07, *p* < .05, which translates to an effect size (*f*) of 0.4, using Lenhard and Lenhard’s (2016) online calculator. The smaller of the two effect sizes (0.4) was selected for power analysis. Based on this, a prior power analysis using G*Power (Faul, Erdfelder, Lang, & Buchner, 2007) indicated that with a power level of .8, an alpha level of .05, and correlations between repeated measures of .5, a sample size of 39 (13 in each group) should be sufficient to reveal an effect of this magnitude. Stöcker and Hoffmann (2004), however, only showed the difference in motor sequence performance between conditions with and without auditory action effects. Whether the difference between conditions with action effects from multiple modalities and from a single modality would show similar magnitude, was not known before the current study. With the sample size used in the study (60 participants), effect sizes (*f*) higher than 0.41 can be detected with a power level of .8, an alpha level of .05, and correlations between repeated measures of .5 by a sensitivity analysis using G*Power (Faul et al., 2007).

Data processing and statistical analysis

Because action effects (tone, rectangle, or both) assigned to key presses were key contingent, an incorrect response always produced the action effect contingent on the pressed key, which deviated from the action effect sequence that the learned motor sequence entailed. Therefore, excessive error rates could lead to participants experiencing a different action effect sequence (Stöcker et al., 2003). To ensure comparability between the action effect sequences that participants within the same group experienced during the experiment, participants with error rates higher than 15% in Phase 4 were excluded and replaced (see Participants section). The error criterion of 15% was in line with Stöcker and Hoffmann (2004).

In Phase 3, error rates were reported as a measurement of sequence acquisition. We compared the error rates for the three action effect groups (auditory, visual, audiovisual) using the nonparametric Kruskal–Wallis test. In Phase 4, the median of ITs and mean IRTs (mean of IRTs within each sequence) computed for each factor combination were analyzed with mixed 3 (group) × 6 (block) × 2 (sequence) ANOVAs, with group as the between-subjects variable. Bonferroni-adjusted post hoc multiple comparisons were performed where there was a main effect of group (there were three pairwise comparisons; thus, *p* values were multiplied by three, with alpha = .05). A trend analysis was performed where there was a significant main effect of block, a significant Group × Block interaction effect, or a significant Sequence × Block interaction effect. Mauchly’s test of sphericity was conducted before all analyses. We used the Greenhouse–Geisser correction to adjust degrees of freedom if the sphericity assumption was violated.

## Results

Practice phase

The Kruskal–Wallis test found that the error rates in the three groups did not differ significantly, *χ*^2^(2) = 1.00, *p* = .61, indicating that there was no difference in sequence acquisition between groups. The mean error rates in the practice phase were 5.7% for the auditory group, 4.7% for the visual group, and 3.6% for the audiovisual group.

Test phase

Initiation times (ITs)

**Fig. 1:**
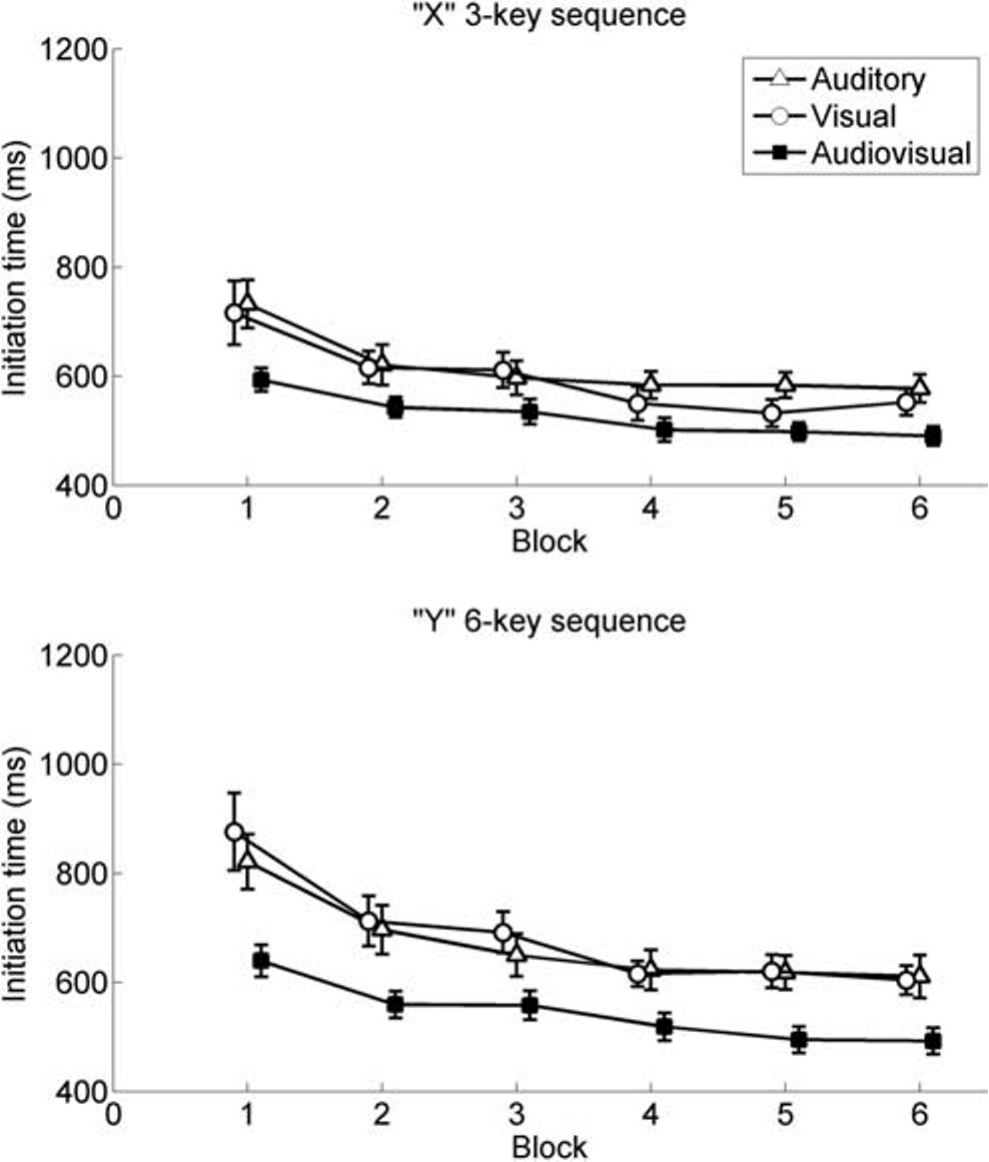
Initiation times for both the “X” short sequence and “Y” long sequence, plotted over blocks in the test phase and divided by action effects. Error bars represent standard errors

The ANOVA revealed a significant main effect of block, *F*(2.29, 130.40) = 57.58, *p* < .001, ${\upeta}_p^2$ = .50. The trend analysis revealed both significant linear, *F*(1, 57) = 82.07, *p* < .001, ${\upeta}_p^2$ = .59, and quadratic, *F*(1, 57) = 66.86, *p* < .001, ${\upeta}_p^2$ = .54, trends, showing that ITs generally decreased with practice, but this effect is asymptotic with the relative decrease in ITs lessening with practice. The main effect of sequence was significant, *F*(1, 57) = 23.64, *p* < .001, ${\upeta}_p^2$ = .29, showing that the ITs in the three-key sequence were shorter than in the six-key sequence. The Block × Sequence interaction was also significant for ITs, *F*(2.42, 138.08) = 4.21, *p* =.012, ${\upeta}_p^2$ = .07. The trend analysis only revealed a significant linear trend, *F*(1, 57) = 8.01, *p* = .006, ${\upeta}_p^2$ = .12, indicating that the difference of ITs between two sequences decreased with practice in a relatively constant, linear fashion. Notably, the main effect of group was significant, *F*(2, 57) = 4.65, *p* = .013, ${\upeta}_p^2$ = .14. Bonferroni-adjusted post hoc multiple comparisons showed that ITs in the audiovisual group were significantly faster than in the other two groups (*p*s < .033). Additionally, the Group × Sequence interaction was significant, *F*(2, 57) = 3.69, *p* = .031, ${\upeta}_p^2$ = .12, showing that the difference of ITs between two sequences was smaller in the audiovisual group than in the other groups (the differences between estimated marginal means for two sequences: auditory group, 74 ms; visual group, 97 ms; audiovisual group, 27 ms). The Group × Block interaction, *F*(4.58, 130.40) = 2.02, *p* = .09, and the three-way Group × Block × Sequence interaction, *F*(4.85, 138.08) = 0.50, *p* = .77, did not approach significance (see Fig. 1).

Mean interresponse times (mean IRTs)

**Fig. 2:**
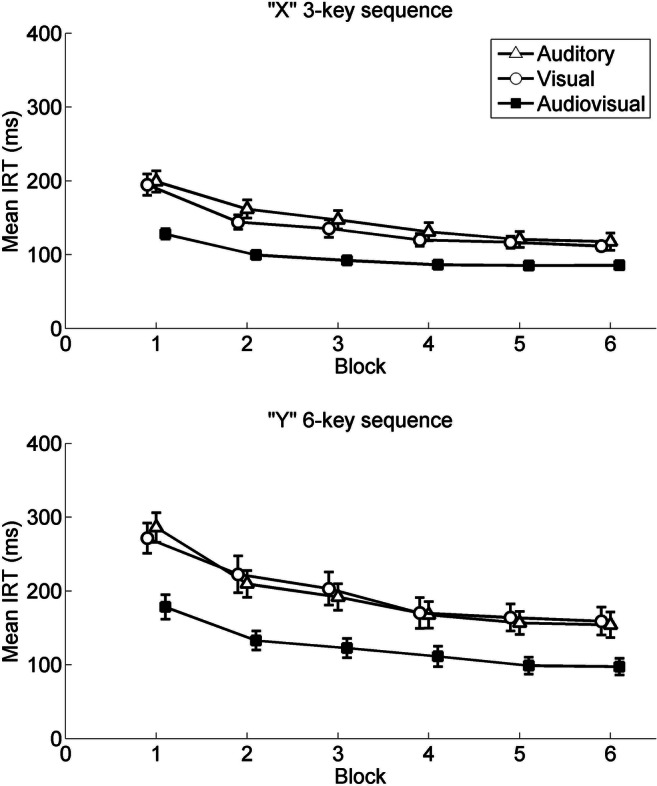
Mean interresponse times for both the “X” short sequence and “Y” long sequence, plotted over blocks in the test phase and divided by action effects. Error bars represent standard errors

The ANOVA showed a significant main effect of block, *F*(2.92, 166.29) = 175.98, *p* < .001,${\upeta}_p^2$ = .76. The trend analysis revealed both significant linear, *F*(1, 57) = 311.93, *p* < .001, ${\upeta}_p^2$ = .85, and quadratic, *F*(1, 57) = 111.02, *p* < .001, ${\upeta}_p^2$ = .66, trends, showing that mean IRTs generally decreased with practice, but this effect is asymptotic, with the relative decrease in mean IRTs lessening with practice. And the main effect of sequence was significant, *F*(1, 57) = 31.68, *p* < .001,${\upeta}_p^2$ = .36, indicating mean IRTs in the three-key sequence were shorter than in the six-key sequence. The Block × Sequence interaction was also significant for mean IRTs, *F*(3.19, 181.82) = 16.93, *p* < .001,${\upeta}_p^2$ = .23. The trend analysis revealed a significant linear trend, *F*(1, 57) = 39.36, *p* < .001, ${\upeta}_p^2$ = .41, showing that the (negative) trend was stronger for the six-key sequence, which resulted in a gradual decrease in the difference of mean IRTs between the two sequences. The significant quadratic trend, *F*(1, 57) = 5.80, *p* = .019, ${\upeta}_p^2$ = .09, indicated that the difference of mean IRTs between the two sequences decreased more notably during early blocks relative to late blocks in the test phase. Notably, the main effect of group was again significant, *F*(2, 57) = 8.22, *p* = .001, ${\upeta}_p^2$ = .22. Bonferroni adjusted post hoc multiple comparisons showed that mean IRTs in the Audiovisual group were significantly faster than in the other two groups (*p*s < .003). Additionally, the Group × Block interaction was significant, *F*(5.84, 166.29) = 4.47, *p* < .001, ${\upeta}_p^2$ = .14. The trend analysis only revealed a significant linear trend, *F*(2, 57) = 8.33, *p* < .001, ${\upeta}_p^2$ = .23, indicating that the difference of mean IRTs between groups decreased with practice in a relatively constant, linear fashion. Neither the interaction of Group × Sequence, *F*(2, 57) = 1.49, *p* = .23, nor three-way Group × Block × Sequence interaction was significant, *F*(6.38, 181.82) = 1.35, *p* = .23^1^ (see Fig. 2).

## Discussion

The present study investigated the contribution of multisensory action effects in motor sequence performance. The task adapted from Stöcker and Hoffmann (2004) was to learn and perform two motor sequences under different action–effect conditions. We observed that compared with the groups with only auditory or visual action effects, there was an advantage in the ITs and mean IRTs performance for the group with audiovisual action effects. These results indicated that action effects with multiple sensory features facilitate the initiation and execution of motor sequences. To our knowledge, this study was the first to investigate the role of action effects on motor sequence performance from multiple sensory modalities perspective. This is an important area, because actions in daily life normally lead to multisensory action effects, including proprioception, vision, and audition, which leads to an activation of a broad network of different modalities (Esser & Haider, 2018).

ITs were significantly faster in the audiovisual group than the other groups, which suggests that motor sequence initiation was improved by providing more action effect features. Stöcker and Hoffmann (2004) showed that ITs were faster in a group with task-irrelevant auditory action effects than without auditory action effects. They argued that task-irrelevant auditory action effects facilitate the chunking of sequence elements into larger units. According to their reasoning, our results for ITs, therefore, indicate that action effects with more features facilitate the associations between successive elements and the further development of motor chunks. The so-called motor chunk is a representation linking a limited number of action elements, such as key presses, together (Klapp, 1995; Sternberg, Monsell, Knoll, & Wright, 1978). As a result, these action elements can be selected as one single action element in a control hierarchy, leading to fast motor sequence initiation (Verwey, 1999). Following the ideomotor principle (Greenwald, 1970), when stimulus–response–effect triplets in the practice phase and response–effect pairs in the test phase are repeatedly experienced in the same order, action effects are first associated with the actions that produced them. Action effects of sequential actions are serially chained, and the associations between consecutive elements of the action–effect sequence are formed (Hoffmann et al., 2001; Stöcker & Hoffmann, 2004; Stöcker et al., 2003). The sequence representations contain not only representations of the actions themselves but also of the action effects associated with those actions (Stöcker & Hoffmann, 2004). It could be speculated that the more action effect features exist, the stronger the associations are between contiguous action effects in the effect sequence, leading to the development of a sequence representation of different quality.

It is worth noting that there was a significant interaction between sequence and group, showing that the sequence-length effect in the audiovisual group was smaller than in the other groups (i.e., the sequence-length effect was reduced by providing more action effect features). The sequence-length effect refers to the fact that the more elements the sequence has, motor sequence initiation takes longer. Reduction of the sequence-length effect is linked with the development of motor chunk (Verwey, 1999). Therefore, the reduction of the sequence length effect might support the chunking-based explanation. However, only one short and one long sequence were used in this study, which differed in several aspects besides their length (e.g. the hand starting the sequence). A more direct approach to control for this in future experiments would be to randomize sequences between participants.

Beyond movement initiation, our results regarding mean IRTs showed that providing more information of action effects from multiple modalities improve motor sequence execution. There are two interpretations based on ideomotor principle that are not mutually exclusive that can explain why this happens. First, as we discussed above, action effects with more features facilitate the development of motor chunks. Chunking also influences motor sequence execution (i.e., mean IRTs). Key presses following a motor chunk initiation can be prepared more easily and are typically fast. This is because these key presses only involve execution processes; these key presses have already been selected and prepared during the initiation of the motor chunk (Abrahamse, Ruitenberg, De Kleine, & Verwey, 2013). Therefore, execution of a motor sequence with a more effective chunking process would be faster. Second, representations of actions include their perceivable sensory action effects and the anticipation of these action effects should activate action control (Kunde, 2001). The more action effect features belong to an action representation, the more action effects are anticipated, which leads to greater activation of action representation (Elsner & Hommel, 2001), which in turn facilitates triggering of each individual key press. The action effects in the audiovisual group contained more effect features, the anticipation of the audiovisual action effects of an individual key press activated the representation of the key press greater, which accelerated the respective key press. Taken together, action effects in the audiovisual group may have reduced mean IRTs in two not mutually exclusive ways, by strengthening the associations between contiguous action effects and by evoking a greater activated representation of each individual key press.

The present design does not rule out potential alternative explanations, however, so that several theoretical possibilities might also account for the benefits from action effects with more features: First, several studies have demonstrated that when actions (e.g., pinches, button presses, tapping on a table) elicit a more reliable, higher quality feedback, action control is more effective (Neszmélyi & Horváth, 2017, 2018, 2019). In our study, the action effects in the audiovisual group might provide more reliable feedback than in other groups, allowing for more efficient motor control (Horváth, Bíró, & Neszmélyi, 2018). Thus, participants in the audiovisual group might be more confident in the success of the actions and can execute not only the subsequent actions within the motor sequence but also all subsequent sequences at a higher speed. Second, the present study measures sequence acquisition by error rates in the practice phase. It might be insensitive towards explicit sequence knowledge. Thus, there might be a difference in explicit sequence knowledge between groups. The evidence for the relevance of action effects for explicit knowledge of the motor sequence has been shown in several studies (Esser & Haider, 2018; Lustig & Haider, 2019; Tubau, Hommel, & López-Moliner, 2007). Tubau et al. (2007) found that response-contingent tone effects facilitate phonetic coding. Phonetic coding in turn increases the likelihood that participants enter a plan-based control mode and enhances the acquisition of explicit knowledge. Introducing visual effects might motivate an imagery-based planning strategy. And the action effects in the Audiovisual group might result in a planning strategy based on both phonetic codes and imagery codes, leading to more explicit knowledge of the motor sequence in the practice phase and better motor sequence performance in the test phase. Third, the beneficial influence of the multisensory action effects could be attributed to stimulus–effect learning. In this study, participants are required to learning the mapping of letters onto keys. The spatially arranged key locations share response–effect compatibility with action effects (Kunde, 2001; Stöcker et al., 2003). The action effects with more features might make it easier to map the required keys onto letters. Fourth, the benefit of multisensory effects might be individual differences. It might be easier for some participants to code auditory action effects into action representations while others might prefer to code the visual action effects. It will make it easier for them if they have a choice to select the modality in which the action effects are coded.

Some caution regarding the generality of the beneficial influence of multisensory action effects are warranted. One the one hand, all action effects in our study were contingently mapped to the response keys in ascending order from left to right. Numerous studies have shown that the impact of action effects on the action depends on the compatibility of the action–effect mapping (e.g., Hoffmann et al., 2001; Kunde, 2001, 2003; Stöcker & Hoffmann, 2004). The compatibility of the action–effect mapping of different modalities should be an important factor for observed ITs and mean IRTs enhancement from unimodal action effect to multisensory action effects. Mutual priming of effect codes would be harmed with a noncorresponding mapping for all modalities (Kunde et al., 2004). The benefits from action effects with more features could then not be found. On the other hand, the relative timing of action effect features from different modalities might another factor for observed ITs and mean IRTs enhancement from unimodal action effect to multisensory action effects. In this study, the auditory action effects (tones) and visual action effects (rectangles) were presented simultaneously in the audiovisual group. However, the processing times for participants were likely to differ for features coded in different modalities and there is (diverging) output latency of the soundcard and the monitor (Zmigrod & Hommel, 2013). There may be a temporal window, within which auditory action effects and visual action effects must fall, for the benefits from multisensory task-irrelevant action effects to motor sequences. With the current data, we cannot suggest the temporal principle and criterion of the beneficial influence of action effects from multiple modalities. Additional experiments are needed to elucidate these points.

Overall, the present study investigated the role of action effects on the motor sequence performance from a multiple-modality perspective. The findings suggest that task-irrelevant action effects from multiple sensory modalities indeed facilitates motor sequence performance more than task-irrelevant from a single sensory modality, leading to faster initiation and execution of motor sequences. One of the most concerning issues in multisensory research is whether information from different modalities is integrated into a coherent representation, or whether information from different sensory modalities is still processed separately. Previous studies have frequently revealed a so-called redundant signals effect (e.g., Diederich & Colonius, 2004; Miller, 1982), in which responses to unimodal (response) stimuli (i.e., preaction) are slower than responses to bimodal stimuli (i.e., when two stimuli from different modalities are presented simultaneously). Two explanations could account for the redundant signals effect: the race model and the coactivation model. According to the race model, a response is triggered by the stimulus detected first, making reaction time to bimodal stimuli faster than to unimodal stimuli by means of “statistical facilitation” (Raab, 1962). However, according to the coactivation model, units of information from different modalities might be integrated first, and the integration of information then triggers the response, which enables a faster response (Miller, 1982). Miller (1982) proposed a race model inequality test to distinguish the race model and the coactivation model. Building on the evidence of performance benefits of multisensory response cues (i.e., preaction), future research could investigate whether the benefits from multisensory task-irrelevant action effects (i.e., postaction) to motor sequences depend on the integration of information from multiple modalities. Furthermore, the research was based on motor sequences; thus, the ecological validity and practical implications of this study are limited. One previous study showed that performance of more complex actions (e.g., ball-tossing) can be primed and enhanced by contingent action effects (Land, 2018). An interesting question to be addressed by future research would be whether action effects with more features better facilitate complex human motor skills, as shown in augmented feedback studies (Effenberg, 2005; Marchal-Crespo, McHughen, Cramer, & Reinkensmeyer, 2010; Sigrist, Rauter, Riener, & Wolf, 2013).

## Supplementary Information

ESM 1 (DOCX 21 kb)

## Footnotes

1 Here, the mean IRTs indicated the mean of all IRTs within each sequence (i.e., two IRTs of the three-key sequence and five IRTs of the six-key sequence). Following the advice of one reviewer, we also recalculated our main analysis of the IRTs data to only include the first two IRTs within each sequence (i.e., the first two IRTs of the three-key sequence and the first two IRTs of the six-key sequence). This proceeding did not alter any of the relevant results of the mean IRTs data (see online supplementary material).
