# Supplementary material for: Multisensory action effects facilitate the performance of motor sequences
Source: Atten Percept Psychophys. 2020 Nov 1;83(1):475–83. doi: 10.3758/s13414-020-02179-9 (PMC7875850; doi:10.3758/s13414-020-02179-9)
Supplement: Supplementary file 1 — (DOCX 21 kb) [file 13414_2020_2179_MOESM1_ESM.docx]

Supplementary material for

**Multisensory Action Effects Facilitate the Performance of Motor Sequences**

Mengkai Luan, Heiko Maurer, Arash Mirifar, Jürgen Beckmann, and Felix Ehrlenspiel

IRT was defined as the interval between two contiguous key presses, as measured from the onset of one key press to the onset of the next. Mean IRTs indicated the mean of all IRTs within each sequence, i.e. two IRTs of the three-key sequence and five IRTs of the six-key sequence. The median of mean IRT computed for each factor combination were analyzed with mixed 3 (Group) × 6 (Block) × 2 (Sequence) ANOVA with Group as the between-subject variable. The ANOVA showed that there was a significant main effect of Sequence, *F*(1, 57) = 31.68, *p* < .001,$\text{η}_{p}^{2}$ = .36, indicating mean IRTs in the three-key sequence were shorter than in the six-key sequence. However, there might be difference in IRTs that are related to sequential position, which caused the observed main effect of Sequence. Following the advice of one reviewer we also re-calculated our main analysis of the IRTs data only including the first two IRTs within each sequence, i.e. the first two IRTs of the three-key sequence and the first two IRTs of the six-key sequence.

In a three-key sequence trial, two IRTs within the three-key sequence were recorded and indicated by $T_{2}$ and $T_{3}$, respectively; in a six-key sequence trial, five IRTs were recorded and indicated by $T_{2}$, $T_{3}$, $T_{4}$, $T_{5}$, and $T_{6}$, respectively. Therefore, only $T_{2}$ and $T_{3}$, which were the first two IRTs within each sequence, were included for our analysis. The median IRTs computed for each factor combination were analyzed with mixed 3 (Group) × 6 (Block) × 2 (Sequence) × 2 (Position: $T_{2}$ and $T_{3}$) ANOVAs with Group as the between-subject variable.

The ANOVA showed a significant main effect of Block, *F*(2.39, 136.35) = 122.70, *p* < .001,$\text{η}_{p}^{2}$ = .68. The trend analysis revealed both significant linear, *F*(1, 57) = 194.20, *p* < .001, $\text{η}_{p}^{2}$ = .77, and quadratic, *F*(1, 57) = 81.02, *p* < .001, $\text{η}_{p}^{2}$ = .59, trends, showing that IRTs ($T_{2}$ and $T_{3}$) generally decreased with practice, but this effect is asymptotic with the relative decrease in IRTs lessening with practice. And the main effect of Sequence was significant, *F*(1, 57) = 32.03, *p* < .001,$\text{η}_{p}^{2}$ = .36, indicating IRTs in the three-key sequence were shorter than in the six-key sequence. The Block × Sequence interaction was also significant for IRTs, *F*(2.66, 151.49) = 23.68, *p* < .001,$\text{η}_{p}^{2}$ = .29. The trend analysis revealed both significant linear, *F*(1, 57) = 39.11, *p* < .001, $\text{η}_{p}^{2}$ = .41, and quadratic, *F*(1, 57) = 21.58, *p* < .001, $\text{η}_{p}^{2}$ = .28, trends, showing that the difference of IRTs between two sequences generally decreased with practice, but this effect is asymptotic with the relative decrease in the difference of IRTs between two sequences lessening with practice. Notably, the main effect of Group was significant, *F*(2, 57) = 8.01, *p* = .001, $\text{η}_{p}^{2}$ = .22. Bonferroni adjusted post-hoc multiple comparisons showed that IRTs in the Audiovisual group were significantly faster than in the other two groups (*p*s < .012). Additionally, the Group × Block interaction was significant, *F*(4.78, 136.35) = 4.74, *p* < .001, $\text{η}_{p}^{2}$ = .14. The trend analysis revealed a significant linear trend, *F*(2, 57) = 6.34, *p* = .003, $\text{η}_{p}^{2}$ = .18, indicating that the difference of mean IRTs between groups decreased with practice in a relatively constant, linear fashion. Neither the interaction of Group × Sequence, *F*(2, 57) = 1.53, *p* = .23, nor three-way Group × Block × Sequence interaction was significant, *F*(5.85, 166.69) = 1.29, *p* = .24. These results showed that the proceeding of only including the first two IRTs within each sequence did not alter any of the relevant results of the mean IRTs (the mean of all IRTs within each sequence) data. Furthermore, the main effect of Position was significant, *F*(1, 57) = 7.35, *p* = .009,$\text{η}_{p}^{2}$ = .11, indicating $T_{3}$ was shorter than $T_{2}$. Most importantly, neither the Group × Position interaction, *F*(2, 57) = 1.87, *p* = .164, nor the Sequence × Position interaction effect, *F*(1, 57) = 0.14, *p* = .72, was not significant, indicating that the difference in IRTs between groups and the difference in IRTs between sequences did not differ for sequential positions.

Our results of mean IRTs showed a significant main effect of sequence, indicating mean IRTs in the three-key sequence were shorter than in the six-key sequence, which seemed to be consistent with the sequence length effect on rate (SLER) found by Sternberg et al. (1978). The SLER refers to mean IRTs increasing as a function of sequence length. However, there were two IRTs within the three-key sequence ($T_{2}$ and $T_{3}$) and five IRTs in a six-key sequence trial ($T_{2}$, $T_{3}$, $T_{4}$, $T_{5}$, and $T_{6}$). There were three more serial positions ($T_{4}$, $T_{5}$, and $T_{6}$) in the six-key sequence trial than the three-key sequence. The serial position effects on individual IRTs could be attributed to the sequence effect on mean IRTs (Rhodes et al., 2004; Sternberg et al., 1978). Therefore, we only include $T_{2}$ and $T_{3}$ for the present analysis. Both sequence trials had corresponding serial positions. The results of $T_{2}$ and $T_{3}$ still showed a significant sequence effect, and this effect was not mediated by the serial position. These results supported the claim that the observed sequence effect on mean IRTs should be primarily attributed to the sequence length.

**References**

Rhodes, B. J., Bullock, D., Verwey, W. B., Averbeck, B. B., & Page, M. P. (2004). Learning and production of movement sequences: Behavioral, neurophysiological, and modeling perspectives. *Human movement science, 23*(5), 699-746.

Sternberg, S., Monsell, S., Knoll, R. L., & Wright, C. E. (1978). The latency and duration of rapid movement sequences: Comparisons of speech and typewriting *Information processing in motor control and learning* (pp. 117-152): Elsevier.
